# Diagnostic and Therapeutic Challenges of Malignant Pleural Mesothelioma

**DOI:** 10.3390/diagnostics12123009

**Published:** 2022-12-01

**Authors:** Jacopo Moro, Simona Sobrero, Carlotta Francesca Cartia, Simona Ceraolo, Roberta Rapanà, Federico Vaisitti, Stefano Ganio, Federica Mellone, Stefano Rudella, Federico Scopis, Danilo La Paglia, Carola Crystel Cacciatore, Enrico Ruffini, Francesco Leo

**Affiliations:** 1 Thoracic Surgery Division, Department of Oncology, San Luigi Gonzaga Hospital Orbassano, University of Turin, 10043 Orbassano, Italy; 2 Department of Thoracic Surgery, Ospedale della Carità Hospital Novara, 28100 Novara, Italy; 3 Nursing Degree Program, Department of Clinical and Biological Sciences, University of Turin, 10124 Torino, Italy; 4 Division of Thoracic Surgery, Department of Surgical Sciences, Città della Salute Hospital Turin, University of Turin, 10126 Torino, Italy

**Keywords:** mesothelioma histopathology, mesothelioma immunohistochemistry, mesothelioma genetics, pleural mesothelial hyperplasia, invasive diagnosis of malignant pleural mesothelioma, atypical mesothelial hyperplasia

## Abstract

Malignant pleural mesothelioma is a rare cancer characterized by a very poor prognosis. Exposure to asbestos is the leading cause of malignant pleural mesothelioma. The preinvasive lesions, the mesothelial hyperplasia and its possible evolution are the focus of the majority of the studies aiming to identify the treatable phase of the disease. The role of BAP-1 and MTAP in the diagnosis of mesothelioma in situ and in the prognosis of malignant pleural mesothelioma is the main topic of recent studies. The management of preinvasive lesions in mesothelioma is still unclear and many aspects are the subject of debate. The diagnosis, the disease staging and the accurate, comprehensive assessment of patients are three key instants for an appropriate management of patients/the disease.

## 1. Introduction

Malignant pleural mesothelioma (MPM) is a rare cancer with a very poor prognosis. It was described for the first time by Klemperer and Rabin [1], who differentiated the localized form of MPM from the diffuse one [2]. The latter is a primary and diffuse tumor of the serosal membranes of the pleura.

According to the SEER database, in 2019, the incidence rate of MPM was 0.7/100.000 person/years, with a difference between the two sexes: 0.3 for females and 1.3 for males. During the last decades, the incidence trends have shown stability in the female population, in contrast to the male one, who registered a peak in 1992 (2.6/100.000 person/years) followed by a progressive decrease in the number of cases [3].

Asbestos represents the major risk factor for mesothelioma; however, a small group of mesotheliomas does not seem to be related to asbestos exposure. Indeed, recent studies on MPM carcinogenesis have highlighted the role of some tumor suppressor genes, such as BAP-1, which are capable of affecting its development, prognosis, and inherited transmission [4].

Currently, the WHO classification recognizes three main subtypes, namely epithelioid, sarcomatous, and biphasic that differ in terms of median survival [5]: 14 months for the epithelioid form and 12 and 3 months for the biphasic and sarcomatous forms, respectively [6]. Therefore, MPM has a very poor prognosis with few therapeutic options.

While many studies focus on preinvasive lesions (mesothelioma in situ), mesothelial hyperplasia and its progression to preinvasive lesions, and to identify the treatable phase of the disease, differentiating between benign and malignant proliferation of cells in the pleura often remains a challenge even for skilled pathologists. An exhaustive diagnosis, disease staging and an accurate, comprehensive assessment based on the patient’s medical history are essential for the correct management of the disease.

By contrast, the diagnosis of preinvasive lesions in mesothelioma in situ (MIS) is still unclear and its management has many aspects that are the subject of debate, mainly because it has recently been recognized as a separate entity, and no guidelines on its treatment have been published yet.

## 2. Genetics and Risk Factors of Malignant Pleural Mesothelioma

Malignant pleural mesothelioma (MPM) is typically detected as an invasive lesion when it/that has already spread in all the pleural layers. The neoplastic transformation of the mesothelial cells is due to the accumulation of genetic alterations, leading to the proliferation of mutated cells.

Asbestos is one of the main risk factors for MPM, although a small group of MPMs seem unrelated to asbestos exposure. Despite MPM being always the same disease, its triggers, the age of onset and prognosis are all features that differ significantly.

### 2.1. Asbestos-Related Mesothelioma

The word “asbestos” derives from an old Greek adjective that means “inextinguishable”. Nowadays, asbestos includes a large group of silicate minerals divided into two major forms in nature: serpentine and amphibolite. The serpentine group consists of chrysolite, whereas the amphibolite group consists of crocidolite, amosite, tremolite, anthophyllite, and actinolite. Both groups can form thin fibers, however, the amphibole fibers are more bio-present in the lung than the serpentine ones.

In recent years, especially in the 1960s and 1970s, chrysolite, amosite and crocidolite were used for industrial, mechanical, and civilian/civic purposes [7,8]. In the 1960s Wagner et al. reported some cases of MPM among miners and their family members in the north-western part of the Cape Province, South Africa, where crocidolite asbestos was mined [9] Even though all types of asbestos are known to influence the development of MPM [10], it is claimed that crocidolite is the most oncogenic form with chrysotile as the least [9,11].

In the past, asbestos was added to many industrial products and widely used in construction because of its physical and chemical properties. In light of the clear relationship between asbestos exposure and MPM development, many European countries banned its use. Unfortunately, some other areas such as Brazil, eastern Europe and many African countries continue to mine and manufacture asbestos products [12,13].

Despite a large number of studies focusing on the relationship between asbestos fibers and pleural and lung cells, the mechanism by which asbestos could contribute to the development of MPM is still not fully understood. Asbestos fibers seem to be able to induce damage in the pleural cells through direct and indirect mechanisms. When inhaled, airborne particles of asbestos can be trapped inside the lungs and translocate to the pleura, as shown by the presence of anthracotic areas (black spots) in the parietal pleura [14].

The effects of asbestos fibers on the body are multiple. After internalization in the pleural cells, they induce the degranulation of lysosomes, as demonstrated by morphological studies [15,16]. In addition, the phagocytosis of asbestos fibers produces intracellular oxidation, which is responsible for damage to the DNA [17]. Moreover, they could interact directly with the mitotic process inducing chromosome disaggregation (abnormal anaphases/telophases) and aneuploidy [17,18], Finally, they stimulate an inflammatory response that produces reactive oxygen and nitrogen species (ROS/RNS) that amplify the DNA damage [19]. Inflammation is related to the increase in TNF-α (tumor necrosis factor α), which triggers the activation of the NF-κB pathway (nuclear factor kappa-light-chain-enhancer of activated B cells), which, in turn, leads to apoptosis resistance and the accumulation of DNA damage. NF-κB determines a dedifferentiation of the epithelial cells and transient-amplifying cells that acquire a cancer stem cell phenotype [20].

Genes such as P16, NF2 (neurofibromatosis 2), and TP53 are typically altered by inflammation induced by asbestos fibers and cause a loss of control of cell proliferation and apoptosis [21]. As shown in other tumors, inflammation promotes and predisposes to the development of cancer and all stages of tumorigenesis, especially chronic inflammation. The significant production of cytokines involved in the inflammatory system leads to the dysregulation of the cell cycle and controlled apoptosis resulting in overproliferation and nonfunctional restoration.

### 2.2. Non Asbestos-Related Mesothelioma

There are other risk factors related to MPM development, such as mineral fibers with a similar structure to asbestos such as erionite and fluoro-edenite. Radiations are also related to different types of cancer; in particular, some studies described the relationship between radiation and malignant mesothelioma in humans.

A rise in MPM cases in workers exposed to radioactive products for a prolonged period of time has been noted, as well as an increased incidence of MPM in patients previously treated with therapeutic radiation for tumors [16,17,18,19,20,21,22,23].

The International Agency for Research on Cancer (IARC) classified multiwalled carbon nanotube 7 (MWCNT-7) as a possible cancerogenic agent for humans. Recent studies have demonstrated intratracheal instillation of MWCNT-7 as a cause of malignant pleural mesothelioma in rats’ lungs [24,25].

Inflammation has always been related to cancer developments [26], but few literature studies underline the relationship between MPM and a chronic serosal inflammatory condition. Accordingly, anecdotal reports have been related to pleural inflammation secondary to therapeutic plombage or chronic empyema with MPM development [27,28,29].

Interleukin 6 (IL-6) has been identified as one of the mediators involved in chronic inflammation which causes MPM development [29,30]. In contrast, despite its role in countless malignancies, smoking does not represent a risk factor. There are some conflicting opinions in the literature regarding the cancerogenic power of simian virus 40 (SM40), so IARC did not classify this agent as cancerogenic in humans [31].

### 2.3. Mesothelioma and BAP-1 Hereditary Cancer Predisposition Syndrome

Recently, the interest in the role of BAP-1 (BRCA1-associated protein–1) in mesothelioma has increased [32,33,34]. BAP-1 is a gene of 9 kilobases that encodes 729 amino acids in 17 exons [32]. It is a nuclear localizing deubiquitinating hydrolase enzyme whose gene is located on chromosome band 3p21. It is a tumor suppressor protein involved in the epigenetic modification of chromatin, in the repair process of damaged DNA; in addition, it regulates apoptosis, cell cycle control, and immune response.

The functional cell signaling system is attributed to the ubiquitin carboxy-terminal hydrolase domain located in the N-terminal region. Other domains of BAP-1 include HCF1 (protein binding motifs for host cell factor 1), BRCA1 FOXk1-k2, YY1, and BRCA1.

One primary role of BAP-1 is the involvement in chromatin modification. In fact, it regulates chromatin architecture by controlling the delicate balance of the histone H2A ubiquitination, a mechanism supposed to be implicated in cancer pathways [32]. Moreover, BAP-1 regulates the response to DNA damage in multiple ways. The DNA damage repair mechanism is carried out through the interaction of the BRCA1/BARD1 complex and BAP-1. The role of BAP-1 is to coordinate the expression of BRCA1, BARD 1, and RAD51 during the homologous recombination of DNA repair in the RAD51-mediated process [33,34].

With regard to the control of cell cycle and cell proliferation, they are regulated by Host Cell Factor 1 (HCF1) [35], which promotes the progression of the cell cycle from the G1 to the S phase. Studies have demonstrated the importance of BAP-1 in this process: the knockdown of BAP-1 at this level could lead to the disruption of the cell cycle at the G1 phase [36].

Along with HCF1 and YY1, BAP-1 is also involved in controlling the expression of the genes involved in cell proliferation [37]. Additionally, recent studies have shown a relationship between BAP-1 and the regulation of apoptosis. As for BAP-1 localized in the endoplasmic reticulum (ER), it is involved in the apoptotic calcium-related pathway, allowing apoptosis to release calcium from the ER to the cytosol [38].

Furthermore, BAP-1 carries out a function in the immune regulation; in fact, research has demonstrated the presence of CD3+ and CD8+ infiltrations in cells affected by uveal melanoma with BAP-1 loss, which is usually related to an increased tumor immune evasion. Therefore, the identification of the loss of BAP-1 expression in some tumors, such as ovarian, lung, and breast carcinomas, has suggested that BAP-1 could be considered a tumor suppressor gene [39].

There are two types of BAP-1 alteration, germline and somatic, and both are related to an increased risk of cancer development [40]. The germline mutation of BAP-1 is an autosomal dominant mutation whose alterations are characterized by missense and frameshift mutation. Three studies have underlined a higher risk of hereditary cancers, such as uveal melanoma, malignant mesothelioma (pleural and peritoneal), renal cell carcinoma and cutaneous melanoma, in patients with a germline mutation of BAP-1 [41]. This type of mutation is typical of “tumor predisposition syndrome” (BAP-1 TPDS). Baumann et al. underlined that patients with BAP-1 TPDS could develop at least one malignancy in 85% of the cases, with a median age of onset of 50 [42].

Malignant pleural mesothelioma, which accounts for 22% of all the neoplasms diagnosed, is the second neoplasm for incidence in tumor predisposition syndrome. The median age at diagnosis is 46 years old, which is lower compared with sporadic malignant pleural mesothelioma [41]. The overall survival of patients with BAP-1 TPDS, on the other hand, is sevenfold longer when compared with those affected by wild-type MPM.

Studies on mice confirmed that a germline BAP-1 mutation is inherited in an autosomal dominant pattern. The BAP-1 mutation in this population poses a high risk of developing MPM and other neoplasms in individuals and affected families [41].

Ohar et al. suggested that mice germline mutation of BAP-1 contributes to increased susceptibility to MPM in asbestos-exposed mice with a mechanism involving a gene-environment interaction [40]. Xu et al. generated BAP-1+/− mice and reported that these mice developed peritoneal malignant mesothelioma at twice the rate of wild-type mice after intraperitoneal injection of crocidolite asbestos [43]. Moreover, MPM occurred earlier in the BAP-1+/− mice. No mesotheliomas were found in BAP-1 +/− mice that had not been exposed to asbestos. Despite contrasting results, Kadariya et al. observed mesothelioma development without asbestos exposure in BAP-1 knockout mice [44].

Regarding the somatic alteration of BAP-1, this appears in similar neoplasms in patients with a germline mutation. Somatic BAP-1 loss is found in 84% of patients with metastatic uveal melanomas. Harbur et al. suggested that somatic mutations of BAP-1 in uveal melanomas predispose to the development of metastases [45]. As already seen in the germline mutations, MPM patients presenting with a somatic mutation of BAP-1 live significantly longer than those affected by wild-type MPM.

In conclusion, BAP-1 is a tumor-suppressor gene whose alteration is related to a higher predisposition to develop MPM, whether the exposure to asbestos is either low or completely absent. The BAP-1 mutation in the germline explains the BAP-1 hereditary cancer predisposition syndrome. Both germline and somatic BAP-1 MPM show a longer survival rate than wild-type MPM [45]. The study of the BAP-1 status in the pleural specimens must be conducted routinely because it allows the diagnosis of mesotheliomas in situ (MIS) [46].

### 2.4. The Role of NF2 and CDKN2A in Malignant Mesothelioma

As has occurred with BAP- 1 in recent years, more genetic alterations in the pathogenesis of malignant mesothelioma have been studied, such as the alteration of ferroptosis and iron-dependent lipid peroxidation [47].

NF2 is a tumor suppressor gene that predisposes patients to the development of bilateral vestibular schwannomas, spinal schwannomas, meningiomas, and malignant mesothelioma [48]. It consists of 17 exons and it is located on chromosome 22q12 that encodes a 595 amino acid protein called *merlin* (moesin-ezrin-radixin-like protein) [49]; the mutation of this gene has been found in 40% of mesotheliomas [50]. Germline and somatic mutations have been associated with meningiomas and schwannomas. In the case of malignant melanomas, only somatic mutations have been identified [51]. The mutations observed in NF2 alterations are nonsense or splice site mutations and frameshift deletions.

NF2 activates the Hippo pathway inhibiting the CRL4-DCAF1 complex, a ubiquitin ligase involved in the degradation of LATS1 and LATS2. Mice exposed to asbestos fibers and inactivating NF2 showed a higher frequency of malignant mesothelioma [52]. Furthermore, NF2 is a ‘gatekeeper’ in mesothelioma induced by asbestos [53]. The expression of delE2 missing exon 2, or delE3 missing exon 3, is associated with tumor development in mice without wild-type NF2.

In malignant mesothelioma, NF2 transcription is usually truncated, and malignant cells cultures show a lack of p16/ARF expression. The Hippo signaling pathway alteration may be implicated in malignant mesothelioma tumorigenesis [52].

The p16 gene belongs to the INK4 family (inhibitor of cyclin-dependent kinase 4a) that inhibits cells growth and works as a tumor suppressor [54,55]. It is located on chromosome 9p21, and the loss of heterozygosis is associated with several malignancies [54]. The p16 genes encode several proteins that regulate the cell cycle, as well as the RB1 and p53 pathways. P16 negatively regulates the pRb-E2F pathway during cell cycle. During cell proliferation, pRb induces phosphorylation of CDK4 and CDK6 at the final stage of the G1 phase through the S phase [56]. Genetic inactivation of p16 is a genetic alteration frequently found in cancer [54]. P16 is very often inactivated in breast cancer (20%) and pancreatic adenocarcinomas (85%) [56]. The overexpression or mutant p16 is recurrently associated with a poor prognosis in neoplastic patients and in numerous malignancies such as neuroblastoma, ovarian, breast and prostate cancers and esophageal squamous cells carcinoma. The genetic alterations are often point mutations homozygous deletion, promoter of hypermethylation, and loss of heterozygosis (LOH). A total of 30% of malignant pleural mesotheliomas related to the p16 genes show one alteration, whether it be deletion, methylation, or point mutation [57].

P16 FISH analysis is essential in biopsy examinations in case of suspected malignant mesothelioma or pleural proliferations [58].

The loss of CDKN2A/p16 function is strongly associated with the development of MPMand along with BAP-1 loss, it should be taken into consideration during cytological evaluation of pleural effusions. This loss of function is mainly associated with sarcomatous mesothelioma [59].

## 3. Mesothelial Hyperplasia and Mesothelioma In Situ

Reactive pleural proliferation is common in multiple processes due to infection, inflammation, pleural effusions, and pulmonary neoplasms.

According to Cagle et al. [60], reactive pleural processes associated with various insults could be considered benign, but they raise the clinical possibility of developing a malignancy over time. These processes should be evaluated during the analysis of the patient’s medical history and the endoscopic, thoracoscopic or laparoscopic examination. The pathologist should think twice before calling a suspicious specimen malignant without having anamnestic evidence or a clear macroscopic picture. Currently, such reactive pleural processes can be differentiated into three main groups: *Typical Mesothelial Hyperplasia* (TMH), *Atypical Mesothelial Hyperplasia* (AMH), and *Mesothelioma* in situ (*MIS*). Mesothelial hyperplasia is found in 15% of pleural specimens.

### 3.1. Typical and Atypical Mesothelial Hyperplasia

From the morphological point of view, normal mesothelial cells are very inconspicuous, flat cells covering the serosal surfaces. In typical mesothelial hyperplasia (*TMH*), a very thin layer of distinctly prominent, flattened to cuboidal cells regularly spaced along the pleural surface can be observed. Such cases of simple hyperplasia do not present cellular atypia, but they show an increased mesothelial thickness associated with an increased growth of the sub-mesothelial connective layer [61]. In more elaborate mesothelial proliferations, various morphological patterns may be observed, with heaped-up aggregates of cells on the surface with or without papillary cores and with multiple degrees of cytologic atypia. The presence of atypia, associated with inflammatory modification, could be present in benign conditions suggested by the patient’s indolent clinical history.

The proliferating mesothelial cells in atypical mesothelial hyperplasia (AMH) generally present with distinctly enlarged nuclei and prominent nucleoli that superficially spread along the pleural surface without stromal invasion. Various degrees of cellular and nuclear atypia may be noticed, but they are mainly confined to areas where the effusion is organized. This ‘pleural organization’ has been described according to distinct organizational patterns and named ‘zonation.’ The two main types of zonation in AMH are:

- A reactive proliferation on the surface or at the edge of an organized effusion;
- A pleural surface entrapped in an inflammatory reaction.

Due to these inflammatory reactions, in certain instances, it can be challenging to determine what an actual stromal invasion is because the proliferating mesothelial cells tend to be trapped in a granulation tissue or even dense fibrous tissue. This is known as ‘entrapment’; even though the focus might appear deep to the free surface, it is not a real stromal invasion, and it could be characterized as a benign lesion. It is important to note here that even though they might appear deeper than the surface, benign proliferations tend to penetrate at the same depth within the normal tissue, unlike MPM, which can invade the tissue without any particular orientation. In addition, elongated capillaries running perpendicular to the pleural surface could be noticed, which are considered benign proliferations compared to the irregular and haphazard vascularity of invasive mesothelioma. Lastly, differently from MPM, necrosis in benign hyperplasia is rarely noticed.

**Figure 1:**
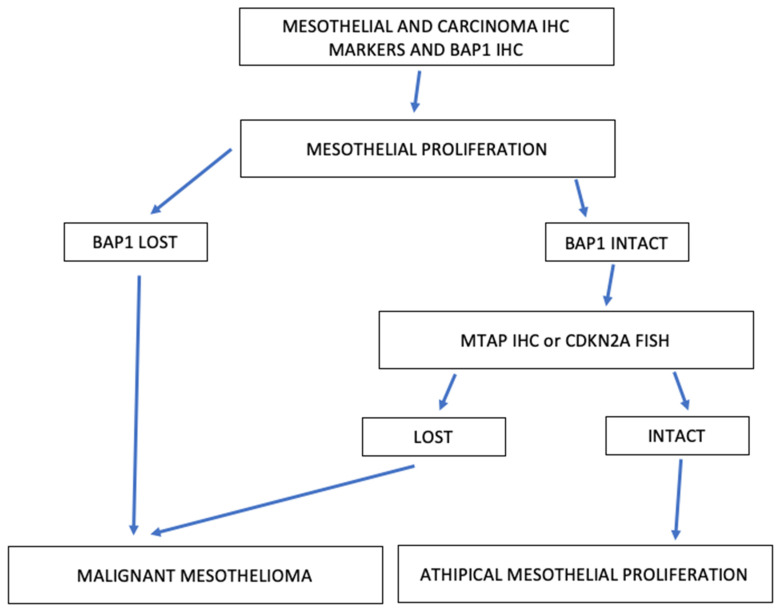
Diagnostic diagram for small biopsy specimens with suspect atypical mesothelial hyperplasia.

The immunohistochemical diagnostic workup for mesothelial proliferations, especially in the cytological evaluation and small specimens, might be very challenging. Immunohistochemical analyses should be used to confirm the mesothelial origin of the biopsies. BAP-1 or MTAP loss or FISH confirmation of CDKN2A homozygosis deletion should confirm malignancy instead of mesothelial proliferation [62] (Figure 1).

### 3.2. Mesothelioma In Situ

The existence of preinvasive lesions in mesothelioma has been hypothesized for a long time. In 2005, Simon et al. described the first mesothelioma in situ in a portion of pleura in which there was a preinvasive lesion and an invasive malignant pleural mesothelioma in the same specimen [63].

The main problem in defining mesothelioma in situ (MIS) is its resemblance to mesothelial hyperplasia. Moreover, at the beginning of 2000, there were no clear and uniform rules distinguishing these two histological entities. The lack of defined criteria was the reason why, at first, MIS was considered to be a different way invasive mesothelioma shows itself rather than a preinvasive lesion [63]. MIS has recently been recognized as a preinvasive lesion that anticipates/precedes the invasive form of pleural mesothelioma. It was formally categorized in the 2020 WHO Blue Book Edition [5]. Clear diagnostic, anatomopathological and immunohistochemical criteria have been established [64,65,66].

In medicine, ‘in situ’ means that the malignant cells do not have an invasive behavior from the histological point of view. According to Churg et al.’s definition, MIS is a superficial proliferation without the invasion of the basal membrane [64,65,66]. His research group has identified the genetic alterations characterizing MIS and the immunohistochemistry markers suitable to locate it [64,65,66].

The mesothelial cells that characterize MIS sometimes show the absence of the BAP-1 nuclear protein [67], an immunohistochemical marker with high specificity (98–100%) for MIS detection but with a lower sensitivity (60–70%).

It is important to note that the WHO diagnostic criteria also include the absence of the nuclear expression of p16, the encoding CDK2 gene, which is better detected using a surrogate of p16 called MTAP [5,6,7,8,9,10,11,12,13,14,15,16,17,18,19,20,21,22,23,24,25,26,27,28,29,30,31,32,33,34,35,36,37,38,39,40,41,42,43,44,45,46,47,48,49,50,51,52,53,54,55,56,57,58,59,60,61,62,63,64], a protein encoded by the homonymous gene situated on chromosome 9, close to the p16 gene. It is a tumor suppressor gene encoding the CDKN2 protein. MTAP is engaged in polyamine metabolism, particularly in the reuptake of adenine and methionine and is often deleted in tandem with the p16 gene due to its proximity [43,44,45,46,47,48,49,50,51,52,53,54,55,56,57,58,59,60,61,62,63,64,65]. MTAP loss is more common in sarcomatous mesothelioma; on the other hand, BAP-1 loss is more common in the epithelioid form of mesothelioma [59].

Morphologically, MIS is a single layer of hyperchromatic discrete mesothelial cells with large nuclei [64] and a picket fence appearance that individually might suggest cytological malignancy. The superficial pleural proliferation can be flat and show either none or minimal cellular atypia, whereas moderate to severe atypia can be observed when small papillary proliferations can be identified. Small biopsy specimens with only a few questionable cells can make the diagnosis even harder.

Regarding the clinical signs, MIS is related to recurrent pleural effusion without radiological and macroscopical features that typically characterize invasive mesothelioma. The presence of MIS must be considered in those patients with recurrent pleural effusion without other evident causes [68].

A diagnosis of MIS cannot be made only by a cytologic evaluation because the assessment of the absence of the stromal invasion could not be demonstrated. Moreover, as Churg observed, it is important to consider that 70% of patients with a ’well-defined mesothelioma in situ’ might develop MPM during the follow-up period, usually within 12–92 months from the diagnosis of MIS [60,68].

## 4. Management

Clinical manifestations of malignant pleural mesothelioma or pleural proliferation are usually non-specific and insidious. The most frequent medical sign is pleural effusion, usually detected on a chest X-ray as a unilateral pleural effusion. Furthermore, if pleural thickening is present, the X-ray should not be the only test used to diagnose MPM.

For accurate disease staging, CT chest scans with intravenous contrast could be very useful [69]. Positron emission tomography (PET-CT) provides functional information on the pleural lesions, especially if prior talc pleurodesis has not been performed yet.

Thoracoscopic biopsy is the gold standard for diagnosis [70]. Pleural biopsy is indicated in recurrent pleural effusions or patients with evidence of pleural lesions at CT or PET-CT scans [69]. It is important to underline that pleural biopsy should confirm all cytological suspicion of malignant pleural mesothelioma.

Video-assisted thoracic surgery (VATS) and medical thoracoscopy play an important role in pleural disease and evacuation of symptomatic pleural effusions. VATS biopsy has a sensitivity of 95%, a specificity of 100% and a negative predictive value of 94% [71].

### 4.1. Surgery

The surgical treatment for MPM consists of two major procedures: extrapleural pneumonectomy and extended pleurectomy/decortication. The former is a massive resection involving an en-bloc resection of the lung, pericardium, diaphragm, and parietal pleura, whereas the latter is a resection of the totality of the parietal and visceral pleura. Extended pleurectomy decortication includes the resection of the pericardium and the hemidiaphragm. These surgical approaches need to be used in association with a systematic lymph node dissection. In addition, since they are characterized by high postoperative mortality and morbidity rates, they can only and exclusively be recommended to very selected patients who have been adequately studied in the pre-operative evaluation.

ERS/ESTS/EACTS/ESTRO guidelines [72] recommend extended pleurectomy/decortication instead of extrapleural pneumonectomy to preserve a good postoperative quality of life as a part of multimodality treatment. Patients with a diagnosis of sarcomatous MPM or prevalent sarcomatous form, N2 disease, and stage IV disease should not be considered for radical surgical resection and should be redirected to other forms of treatment.

There is no international agreement on recommendations and guidelines on the surgical approach to MPM due to a lack of randomized clinical trials. MPM surgery is commonly considered only as cytoreductive [73], and not as a radical solution as is the case with other solid tumors. Moreover, it is seen as part of a multimodal treatment therapy. Despite a lack of inclusion criteria, the patients’ selection for surgical treatment plays an important role in affecting the degree of surgical success and in low morbidity and mortality rates [74].

### 4.2. Radiotherapy

Palliative radiotherapy could be a good choice in the treatment of a painful disease site infiltrated with MPM.

Numerous randomized controlled trials investigating radiotherapy on drain site or trocar access to prevent malignant mesothelioma seeding showed contrasting results [72].

### 4.3. Medical Treatment

The medical treatment for malignant pleural mesothelioma has remained the same since 2009. The first line of therapy is still platinum-based chemotherapy and pemetrexed associated with folic acid and vitamin B12 supplementation [72]. Chemotherapy should be started before clinical deterioration. The Brims score defined four risk groups, and the major risk factor was weight loss [75,76].

Mesothelioma Avastin Cisplatin Pemetrexed Study (MAPS) showed a significantly longer survival in patients who were administered bevacizumab in addition to platinum-based chemotherapy and pemetrexed. MAPS trial also showed a free-progression survival of 2 months in patients who had received bevacizumab [77].

BAP-1 loss in MPM seems sensitive to EZH2 target therapy [78]. CAR T-cell and gene therapy trials are ongoing, and in the future, there could be new therapies for treating MPM as first-line therapy and salvage therapy.

Multimodal treatment with macroscopic complete surgical resection and platinum pemetrexed-based doublet therapy records higher overall survival than a single treatment. Despite that, it is characterized by increased treatment-related morbidity and mortality [79].

New immunotherapy treatments have been introduced since 2009. Immune checkpoint inhibitors have been tested for years. Inhibitors such as anti-CTLA-4 (tremelimumab, ipilimumab), anti-PD-1 (nivolumab, pembrolizumab) and anti-PD-L1 (avelumab, dorvalumab) are being tested in clinical trials. CheckMate 743, an open-label, randomized, phase III clinical trial, tested the efficacy of nivolumab and ipilimumab as a first-line therapy versus platinum-based and pemetrexed chemotherapy for unresectable MPM. Results reported an improvement in the overall survival with immunotherapy compared to standard-of-care (platinum plus pemetrexed). In addition, the trial showed a 2-year overall survival of 41% vs. 27%, and the benefits of immunotherapy were demonstrated in all the subgroups, except for the one with patients older than 75. The median follow-up between epithelioid and non-epithelioid histology showed a survival improvement in both subgroups who were administered the nivolumab and ipilimumab therapy. Based upon the CheckMade 743 trial results, the US Food and Drug Administration approved nivolumab plus ipilimumab for patients diagnosed with unresectable MPM [80].

The MiST2 trial, a single-arm, open-label phase 2 trial, tested abemaciclib, an orally bioavailable CDK4 and CDK6 inhibitor, in patients with p16ink4A deficit and a radiological progression after platinum-based chemotherapy. Results demonstrated a 12-week disease control rate of 54%, with 80% of the patients showing a reduction in tumor volume [81].

The precise location of MPM in the patient needs to be known in order to recommend the best treatment modality for a disease characterized by a very high 5-year mortality rate.

## 5. Talc: The Potential Link between Mesothelial Hyperplasia and Mesothelioma In Situ

It is well known and accepted that chemical pleurodesis with talc insufflation is a therapeutic procedure that could be recommended for patients with symptomatic recurrent pleural effusion.

As already said, VATS procedure is a good management choice to evacuate the pleural space, make a diagnosis, and proceed with pleurodesis, a palliative treatment that prevents the new recurrence of effusion in malignant pleural effusions. The two major contraindications to pleurodesis are the impossibility of removing all the pleural fluid due to the multiple loculation of the pleural space and the ‘trapped lung’, an unexpandable lung [82]. The goal is to create a symphysis between the parietal and visceral pleura that can prevent recurrence of effusion. Insufflation of talcum powder into the pleural cavity produces a significant inflammatory reaction leading to an organized fibrinous pleuritis over time. The mesothelial cells play a key role in this phase.

**Figure 2:**
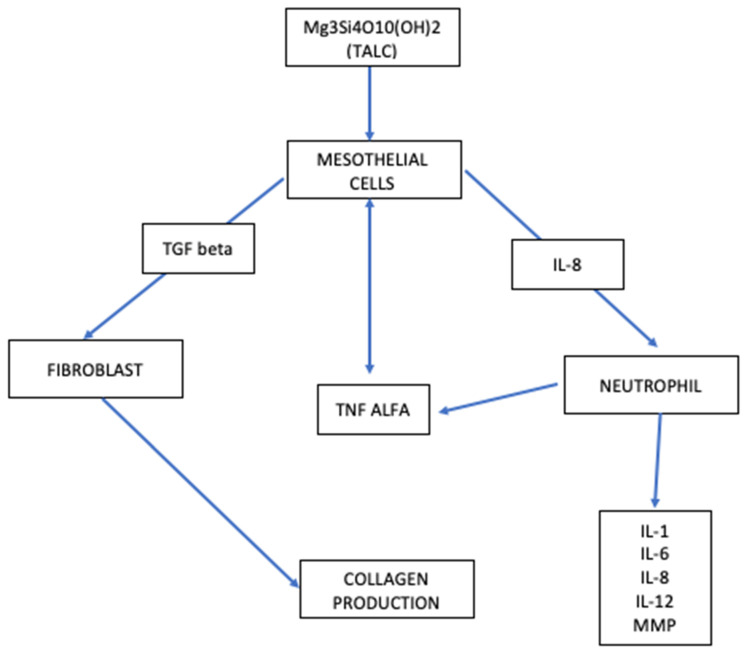
Interaction between mesothelial cells and talc powder [82].

The success of chemical pleurodesis is secondary to the significant damage to the mesothelium surface, inducing the inflammatory cascade (Figure 2) suitable to produce collagen fibers that will lead to the pleural symphysis. The activation of the inflammation cascade occurs through the production of chemokines by damaged mesothelial cells, such as interleukin 8, TGF beta (transforming growth factor beta), MCP1 (monocyte chemoattractive protein 1), TNF alpha (tumor necrosis factor-alpha), VEGF (vascular endothelial growth factor), PDGF (platelet-derivate growth factor) and bFGF (basic fibroblast growth factor) [82]. At the same time, there is a balance in the activation of fibrinogenesis and fibrinolysis. Angiogenesis is also balanced between stimulus and inhibition since the angiogenetic stimulation should increase pleural fluid formation and consequently make pleurodesis ineffective.

Inflammation plays an important role in chemical pleurodesis. The talc stimulates a granulomatous and histiocytic reaction to a foreign body. This reaction was confirmed by an animal model in which talc pleurodesis was inefficient when nonsteroidal anti-inflammatory drugs were administered [83].

It is well known in the literature that chronic inflammation stimulates the pro-oncogenetic pathway and plays an important role in tumorigenesis and all-stage tumor development. The activation of interleukins 6, 17, and 11 can increase the proliferation of tumor cells, especially in particular conditions such as hypoxia and lack of nutrients [84]. Calon et al. showed that interleukin 11 activates fibroblast tumor necrosis factor beta, triggering tumor invasion and immune escape in rectal colon cancer [84,85].

All these mechanisms are more important in the evolution of MIS and in a possible neoplastic transformation of mesothelial hyperplasia, especially the atypical one.

While malignant pleural mesothelioma treatments are well-described and supported by guidelines, this is not the same for mesothelioma in situ and mesothelial hyperplasia. When MIS is considered a malignant disease but still not invasive, the WHO classification adopts the term atypical mesothelial hyperplasia (AMH) for a benign proliferation that might or might not become malignant over time [5].

Talc pleurodesis is a well-accepted palliative treatment of MPM; however, it could trigger the evolution of clearly malignant diseases such as MIS and AMH. Indeed, its use for the treatment of MIS and mesothelial hyperplasia is still the subject of debate. In these cases, an accurate multidisciplinary discussion could help in making the best treatment choice. Blintcliffe et al. suggested that pleurodesis may be the last choice in benign pleural or suspected malignant effusion treated with an indwelling pleural catheter without observed benefits [86]. At the same time, in vitro observations suggest that maintaining an ongoing pleural effusion may help cancer cells to survive and develop [87,88].

Chronic inflammation seems to trigger the development of peritoneal mesothelioma in patients with endometriosis and this could be the same in the pleural cavity with persistent talc inflammation.

The progression time described for MIS ranges from 12 to 90 months with a median time of 5 years [60]. In contrast, no study describes the latency time of neoplastic evolution of AMH. In the future, further prospective studies are needed to better understand the evolution or progression of these pleural entities.

## 6. Conclusions

Malignant pleural mesothelioma is a rare disease characterized by a high 5-year mortality rate. Looking at patients’ therapeutic chances, some progress has been made during the past years; however, the prognosis remains gloomy.

Many aspects regarding its preinvasive forms, such as MIS and AMH, are still unclear. In particular, the management of MIS has many aspects that are the subject of debate, and there are no clear indications for proper follow-up in AMH. For this reason, in order to define appropriate management, the focus of future prospective studies should be a better knowledge of all the preinvasive lesions and their evolution into malignancy. The sea is still stormy for patients with malignant pleural mesothelioma, but further research could be a lifeline.

## References

[1] Nakas A., Martin-Ucar A.E., Edwards J.G., Waller D.A. (2008). Localised malignant pleural mesothelioma: A separate clinical entity requiring aggressive local surgery. Eur. J. Cardio-Thoracic Surg..

[2] Ribak J., Lilis R., Suzuki Y., Penner L., Selikoff I.J. (1988). Malignant mesothelioma in a cohort of asbestos insulation workers: Clinical presentation, diagnosis, and causes of death. Occup. Environ. Med..

[3] SEER Database. https://seer.cancer.gov.

[4] Testa J.R., Cheung M., Pei J., Below J.E., Tan Y., Sementino E., Cox N.J., Dogan A.U., Pass H.I., Trusa S. (2012). Germline BAP1 mutations predispose to malignant mesothelioma. Nat. Genet..

[5] WHO (2021). Classification of Tumors, 5th Edition-Cap-Tumor of Pleura e Pericardium.

[6] Meyerhoff R.R., Yang C.-F.J., Speicher P.J., Gulack B.C., Hartwig M.G., D’Amico T.A., Harpole D.H., Berry M.F. (2015). Impact of mesothelioma histologic subtype on outcomes in the Surveillance, Epidemiology, and End Results database. J. Surg. Res..

[7] Yang H., Testa J.R., Carbone M. (2008). Mesothelioma Epidemiology, Carcinogenesis, and Pathogenesis. Curr. Treat. Options Oncol..

[8] de Assis L., Isoldi M.C. (2013). The function, mechanisms, and role of the genes PTEN and TP53 and the effects of asbestos in the development of malignant mesothelioma: A review focused on the genes’ molecular mechanisms. Tumor Biol..

[9] Wagner J.C., Sleggs C.A., Marchand P. (1960). Diffuse Pleural Mesothelioma and Asbestos Exposure in the North Western Cape Province. Occup. Environ. Med..

[10] Magnani C., Fubini B., Mirabelli D., Bertazzi P.A., Bianchi C., Chellini E., Gennaro V., Marinaccio A., Menegozzo M., Merler E. (2013). Pleural mesothelioma: Epidemiological and public health issues. Report from the Second Italian Consensus Conference on Pleural Mesotheli-oma. Med. Lav..

[11] McDonald A.D., McDonald J. (1978). Mesothelioma after crocidolite exposure during gas mask manufacture. Environ. Res..

[12] Tan C., Treasure T. (2005). Mesothelioma: Time to take stock. J. R. Soc. Med..

[13] LaDou J. (2004). The asbestos cancer epidemic. Environ. Health Perspect..

[14] Boutin C., Dumortier P., Rey F., Viallat J.R., De Vuyst P. (1996). Black spots concentrate oncogenic asbestos fibers in the parietal pleura. Thoracoscopic and mineralogic study. Am. J. Respir. Crit. Care Med..

[15] Jaurand M.C., Kaplan H., Thiollet J., Pinchon M.C., Bernaudin J.F., Bignon J. (1979). Phagocytosis of chrysotile fibers by pleural mesothelial cells in culture. Am. J. Pathol..

[16] Jaurand M.-C., Fleury-Feith J. (2005). Pathogenesis of malignant pleural mesothelioma. Respirology.

[17] Liu W., Ernst J.D., Broaddus V.C. (2000). Phagocytosis of Crocidolite Asbestos Induces Oxidative Stress, DNA Damage, and Apoptosis in Mesothelial Cells. Am. J. Respir. Cell Mol. Biol..

[18] Yegles M., Saint-Etienne L., Renier A., Janson X., Jaurand M.-C. (1993). Induction of Metaphase and Anaphase/Telophase Abnormalities by Asbestos Fibers in Rat Pleural Mesothelial Cells In Vitro. Am. J. Respir. Cell Mol. Biol..

[19] Shukla A., Gulumian M., Hei T.K., Kamp D., Rahman Q., Mossman B.T. (2003). Multiple roles of oxidants in the pathogenesis of asbestos-induced diseases. Free Radic. Biol. Med..

[20] Yang H., Bocchetta M., Kroczynska B., Elmishad A.G., Chen Y., Liu Z., Bubici C., Mossman B.T., Pass H.I., Testa J.R. (2006). TNF-α inhibits asbestos-induced cytotoxicity via a NF-κB-dependent pathway, a possible mechanism for asbestos-induced oncogenesis. Proc. Natl. Acad. Sci. USA.

[21] Bueno R., Stawiski E.W., Goldstein L.D., Durinck S., De Rienzo A., Modrusan Z., Gnad F., Nguyen T.T., Jaiswal B.S., Chirieac L.R. (2016). Comprehensive genomic analysis of malignant pleural mesothelioma identifies recurrent mutations, gene fusions and splicing alterations. Nat. Genet..

[22] Li X., Brownlee N.A., Sporn T.A., Mahar A., Roggli V.L. (2015). Malignant (Diffuse) Mesothelioma in Patients With Hematologic Malignancies: A Clinicopathologic Study of 45 Cases. Arch. Pathol. Lab. Med..

[23] Schubauer-Berigan M.K., Daniels R.D., Bertke S.J., Tseng C.-Y., Richardson D.B. (2015). Cancer Mortality through 2005 among a Pooled Cohort of U.S. Nuclear Workers Exposed to External Ionizing Radiation. Radiat. Res..

[24] Xu J., Futakuchi M., Shimizu H., Alexander D.B., Yanagihara K., Fukamachi K., Suzui M., Kanno J., Hirose A., Ogata A. (2012). Multi-walled carbon nanotubes translocate into the pleural cavity and induce visceral mesothelial proliferation in rats. Cancer Sci..

[25] Fukushima S., Kasai T., Umeda Y., Ohnishi M., Sasaki T., Matsumoto M. (2018). Carcinogenicity of multi-walled carbon nanotubes: Challenging issue on hazard assessment. J. Occup. Health.

[26] Greten F.R., Grivennikov S.I. (2019). Inflammation and Cancer: Triggers, Mechanisms, and Consequences. Immunity.

[27] Kodama Y., Hoshi S., Minami M., Kiso M., Takezawa T., Arai T., To Y., Teshima S., Suzuki N. (2008). Malignant mesothelioma associated with chronic em-pyema with elevation of serum CYFRA19: A case report. Biosci. Trends.

[28] Roviaro G.C., Sartori F., Calabrò F., Varoli F. (1982). The association of pleural mesothelioma and tuberculosis. Am. Rev. Respir. Dis..

[29] Attanoos R.L., Churg A., Galateau-Salle F., Gibbs A.R., Roggli V.L. (2018). Malignant Mesothelioma and Its Non-Asbestos Causes. Arch. Pathol. Lab. Med..

[30] Dostert C., Pétrilli V., Van Bruggen R., Steele C., Mossman B.T., Tschopp J. (2008). Innate Immune Activation Through Nalp3 Inflammasome Sensing of Asbestos and Silica. Science.

[31] International Agency for Cancer Research (IARC) (2014). IARC Monographs–Malaria and Some Polyomaviruses (SV40, BK, JC, and Mekel Cell Viruses), Volume 104. Reviews of Human Car-cinogens. IARC Monographs on the Evaluation of Carcinogenic Risks to Humans.

[32] Louie B.H., Kurzrock R. (2020). BAP1: Not just a BRCA1-associated protein. Cancer Treat. Rev..

[33] Ismail I.H., Davidson R., Gagné J.-P., Xu Z.Z., Poirier G.G., Hendzel M.J. (2014). Germline Mutations in BAP1 Impair Its Function in DNA Double-Strand Break Repair. Cancer Res..

[34] Yu H., Pak H., Hammond-Martel I., Ghram M., Rodrigue A., Daou S., Barbour H., Corbeil L., Hébert J., Drobetsky E. (2013). Tumor suppressor and deubiquitinase BAP1 promotes DNA double-strand break repair. Proc. Natl. Acad. Sci. USA.

[35] Machida Y.J., Machida Y., Vashisht A.A., Wohlschlegel J.A., Dutta A. (2009). The Deubiquitinating Enzyme BAP1 Regulates Cell Growth via Interaction with HCF-1. J. Biol. Chem..

[36] Pan H., Jia R., Zhang L., Xu S., Wu Q., Song X., Zhang H., Ge S., Xu X.L., Fan X. (2015). BAP1 regulates cell cycle progression through E2F1 target genes and mediates transcriptional silencing via H2A monoubiquitination in uveal melanoma cells. Int. J. Biochem. Cell Biol..

[37] Yu H., Mashtalir N., Daou S., Hammond-Martel I., Ross J., Sui G., Hart G.W., Rauscher F.J., Drobetsky E., Milot E. (2010). The Ubiquitin Carboxyl Hydrolase BAP1 Forms a Ternary Complex with YY1 and HCF-1 and Is a Critical Regulator of Gene Expression. Mol. Cell. Biol..

[38] Bononi A., Giorgi C., Patergnani S., Larson D., Verbruggen K., Tanji M., Pellegrini L., Signorato V., Olivetto F., Pastorino S. (2017). BAP1 regulates IP3R3-mediated Ca2+ flux to mitochondria suppressing cell transformation. Nature.

[39] Quetel L., Meiller C., Assié J., Blum Y., Imbeaud S., Montagne F., Tranchant R., De Wolf J., Caruso S., Copin M. (2020). Genetic alterations of malignant pleural mesothelioma: Association with tumor heterogeneity and overall survival. Mol. Oncol..

[40] Ohar J.A., Cheung M., Talarchek J., Howard S.E., Howard T.D., Hesdorffer M., Peng H., Rauscher F.J., Testa J.R. (2016). Germline BAP1 Mutational Landscape of Asbestos-Exposed Malignant Mesothelioma Patients with Family History of Cancer. Cancer Res..

[41] Haugh A.M., Njauw C.-N., Bubley J.A., Verzì A.E., Zhang B., Kudalkar E., Vandenboom T., Walton K., Swick B., Kumar R. (2017). Genotypic and Phenotypic Features of BAP1 Cancer Syndrome: A Report of 8 New Families and Review of Cases in the Literatur. JAMA Dermatol..

[42] Baumann F., Flores E., Napolitano A., Kanodia S., Taioli E., Pass H., Yang H., Carbone M. (2014). Mesothelioma patients with germline BAP1 mutations have 7-fold improved long-term survival. Carcinogenesis.

[43] Xu J., Kadariya Y., Cheung M., Pei J., Talarchek J., Sementino E., Tan Y., Menges C.W., Cai K.Q., Litwin S. (2014). Germline Mutation of *Bap1* Accelerates Development of Asbestos-Induced Malignant Mesothelioma. Cancer Res..

[44] Kadariya Y., Cheung M., Xu J., Pei J., Sementino E., Menges C.W., Cai K.Q., Rauscher F.J., Klein-Szanto A.J., Testa J.R. (2016). Bap1 Is a Bona Fide Tumor Suppressor: Genetic Evidence from Mouse Models Carrying Heterozygous Germline *Bap1* Mutations. Cancer Res..

[45] Harbour J.W., Onken M.D., Roberson E.D.O., Duan S., Cao L., Worley L.A., Council M.L., Matatall K.A., Helms C., Bowcock A.M. (2010). Frequent Mutation of BAP1 in Metastasizing Uveal Melanomas. Science.

[46] Cigognetti M., Lonardi S., Fisogni S., Balzarini P., Pellegrini V., Tironi A., Bercich L., Bugatti M., Rossi G., Murer B. (2015). BAP1 (BRCA1-associated protein 1) is a highly specific marker for differentiating mesothelioma from reactive mesothelial proliferations. Mod. Pathol..

[47] Wu J., Minikes A.M., Gao M., Bian H., Li Y., Stockwell B.R., Chen Z.-N., Jiang X. (2019). Intercellular interaction dictates cancer cell ferroptosis via NF2–YAP signalling. Nature.

[48] Bachir S., Shah S., Shapiro S., Koehler A., Mahammedi A., Samy R.N., Zuccarello M., Schorry E., Sengupta S. (2021). Neurofibromatosis Type 2 (NF2) and the Implications for Vestibular Schwannoma and Meningioma Pathogenesis. Int. J. Mol. Sci..

[49] Evans D.G.R. (2009). Neurofibromatosis type 2 (NF2): A clinical and molecular review. Orphanet J. Rare Dis..

[50] Bianchi A.B., I Mitsunaga S., Cheng J.Q., Klein W.M., Jhanwar S.C., Seizinger B., Kley N., Klein-Szanto A.J., Testa J.R. (1995). High frequency of inactivating mutations in the neurofibromatosis type 2 gene (NF2) in primary malignant mesotheliomas. Proc. Natl. Acad. Sci. USA.

[51] Thurneysen C., Opitz I., Kurtz S., Weder W., Stahel R.A., Felley-Bosco E. (2009). Functional inactivation of NF2/merlin in human mesothelioma. Lung Cancer.

[52] Guo G., Chmielecki J., Goparaju C., Heguy A., Dolgalev I., Carbone M., Seepo S., Meyerson M., Pass H.I. (2015). Whole-Exome Sequencing Reveals Frequent Genetic Alterations in *BAP1*, *NF2*, *CDKN2A*, and *CUL1* in Malignant Pleural Mesothelioma. Cancer Res..

[53] Kinzler K.W., Vogelstein B. (1997). Gatekeepers and caretakers. Nature.

[54] Serrano M. (1997). The Tumor Suppressor Protein p16INK4a. Exp. Cell Res..

[55] Komata T., Kanzawa T., Takeuchi H., Germano I.M., Schreiber M., Kondo Y., Kondo S. (2003). Antitumour effect of cyclin-dependent kinase inhibitors (p16INK4A, p18INK4C, p19INK4D, p21WAF1/CIP1 and p27KIP1) on malignant glioma cells. Br. J. Cancer.

[56] Li J., Poi M.J., Tsai M.-D. (2011). Regulatory Mechanisms of Tumor Suppressor P16^INK4A^ and Their Relevance to Cancer. Biochemistry.

[57] Hirao T., Bueno R., Chen C.-J., Gordon G.J., Heilig E., Kelsey K.T. (2002). Alterations of the p16INK4 locus in human malignant mesothelial tumors. Carcinogenesis.

[58] Marshall K., Jackson S., Jones J., Holme J., Lyons J., Barrett E., Taylor P., Bishop P., Hodgson C., Green M. (2020). Homozygous deletion of CDKN2A in malignant mesothelioma: Diagnostic utility, patient characteristics and survival in a UK mesothelioma centre. Lung Cancer.

[59] Okazaki Y., Misawa N., Akatsuka S., Kohyama N., Sekido Y., Takahashi T., Toyokuni S. (2020). Frequent homozygous deletion of *Cdkn2a/2b* in tremolite-induced malignant mesothelioma in rats. Cancer Sci..

[60] Cagle P.T., Churg A. (2005). Differential Diagnosis of Benign and Malignant Mesothelial Proliferations on Pleural Biopsies. Arch. Pathol. Lab. Med..

[61] Savic I., Myers J. (2021). Update on Diagnosing and Reporting Malignant Pleural Mesothelioma. Acta Med. Acad..

[62] Dacic S., Roy S., Lyons M.A., von der Thusen J.H., Galateau-Salle F., Churg A. (2020). Whole exome sequencing reveals BAP1 somatic abnormalities in mesothelioma in situ. Lung Cancer.

[63] Simon F., Johnen G., Krismann M., Müller K.-M. (2005). Chromosomal alterations in early stages of malignant mesotheliomas. Virchows Arch..

[64] Pulford E., Henderson D.W., Klebe S. (2020). Malignant mesothelioma in situ: Diagnostic and clinical considerations. Pathology.

[65] Churg A., Hwang H., Tan L., Qing G., Taher A., Tong A., Bilawich A., Dacic S. (2018). Malignant mesothelioma in situ. Histopathology.

[66] Beasley M.B., Galateau-Salle F., Dacic S. (2021). Pleural mesothelioma classification update. Virchows Arch..

[67] Berg K.B., Dacic S., Miller C., Cheung S., Churg A. (2018). Utility of Methylthioadenosine Phosphorylase Compared With BAP1 Immunohistochemistry, and CDKN2A and NF2 Fluorescence In Situ Hybridization in Separating Reactive Mesothelial Proliferations From Epithelioid Malignant Mesotheliomas. Arch. Pathol. Lab. Med..

[68] Churg A., Salle F.G., Roden A.C., Attanoos R., Von Der Thusen J.H., Tsao M., Chang N., De Perrot M., Dacic S. (2019). Malignant mesothelioma in situ: Morphologic features and clinical outcome. Mod. Pathol..

[69] Sharif S., Zahid I., Routledge T., Scarci M. (2011). Does positron emission tomography offer prognostic in-formation in malignant pleural mesothelioma?. Interact. Cardiovasc. Thorac. Surg..

[70] Zahid I., Sharif S., Routledge T., Scarci M. (2011). What is the best way to diagnose and stage malignant pleural mesothelioma?. Interact. Cardiovasc. Thorac. Surg..

[71] Bueno R., Opitz I., IASLC Mesothelioma Taskforce (2018). Surgery in Malignant Pleural Mesothelioma. J. Thorac. Oncol..

[72] Scherpereel A., Opitz I., Berghmans T., Psallidas I., Glatzer M., Rigau D., Astoul P., Bölükbas S., Boyd J., Coolen J. (2020). ERS/ESTS/EACTS/ESTRO guidelines for the management of malignant pleural mesothelioma. Eur. Respir. J..

[73] Sugarbaker D.J. (2006). Macroscopic complete resection: The goal of primary surgery in multimo-dality therapy for pleural mesothelioma. J. Thorac. Oncol..

[74] Opitz I., Furrer K. (2020). Preoperative Identification of Benefit from Surgery for Malignant Pleural Mesothelioma. Thorac. Surg. Clin..

[75] Buikhuisen W., Hiddinga B.I., Baas P., van Meerbeeck J. (2015). Second line therapy in malignant pleural mesothelioma: A systematic review. Lung Cancer.

[76] Brims F.J.H., Meniawy T.M., Duffus I., de Fonseka D., Segal A., Creaney J., Maskell N., Lake R.A., de Klerk N., Nowak A.K. (2016). A Novel Clinical Prediction Model for Prognosis in Malignant Pleural Mesothelioma Using Decision Tree Analysis. J. Thorac. Oncol..

[77] Zalcman G., Mazieres J., Margery J., Greillier L., Audigier-Valette C., Moro-Sibilot D., Molinier O., Corre R., Monnet I., Gounant V. (2015). Bevacizumab for newly diagnosed pleural mesothelioma in the Mesothelioma Avastin Cisplatin Pemetrexed Study (MAPS): A randomised, controlled, open-label, phase 3 trial. Lancet.

[78] LaFave L.M., Béguelin W., Koche R., Teater M., Spitzer B., Chramiec A., Papalexi E., Keller M.D., Hricik T., Konstantinoff K. (2015). Loss of BAP1 function leads to EZH2-dependent transformation. Nat. Med..

[79] De Bondt C., Psallidas I., Van Schil P.E.Y., van Meerbeeck J.P. (2018). Combined modality treatment in mesothelioma: A systemic literature review with treatment recommendations. Transl. Lung Cancer Res..

[80] Baas P., Scherpereel A., Nowak A.K., Fujimoto N., Peters S., Tsao A.S., Mansfield A.S., Popat S., Jahan T., Antonia S. (2021). First-line nivolumab plus ipilimumab in unresectable malignant pleural mesothelioma (CheckMate 743): A multicentre, randomised, open-label, phase 3 trial. Lancet.

[81] A Fennell D., King A., Mohammed S., Greystoke A., Anthony S., Poile C., Nusrat N., Scotland M., Bhundia V., Branson A. (2022). Abemaciclib in patients with p16ink4A-deficient mesothelioma (MiST2): A single-arm, open-label, phase 2 trial. Lancet Oncol..

[82] Mierzejewski M., Korczynski P., Krenke R., Janssen J.P. (2019). Chemical pleurodesis–a review of mechanisms involved in pleural space obliteration. Respir. Res..

[83] Kaya S.O., Bir F., Atalay H., Onem G., Aytekin F.O., Saçar M. (2005). Effect of Diclofenac on Experimental Pleurodesis Induced by Tetracycline in Rabbits. J. Investig. Med..

[84] Calon A., Lonardo E., Berenguer-Llergo A., Espinet E., Hernando-Momblona X., Iglesias M., Sevillano M., Palomo-Ponce S., Tauriello D.V.F., Byrom D. (2015). Stromal gene expression defines poor-prognosis subtypes in colorectal cancer. Nat. Genet..

[85] Calon A., Espinet E., Palomo-Ponce S., Tauriello D.V.F., Iglesias M., Céspedes M.V., Sevillano M., Nadal C., Jung P., Zhang X.H.-F. (2012). Dependency of Colorectal Cancer on a TGF-β-Driven Program in Stromal Cells for Metastasis Initiation. Cancer Cell.

[86] Bintcliffe O.J., Lee G.Y., Rahman N.M., Maskell N.A. (2016). The management of benign non-infective pleural effusions. Eur. Respir. Rev..

[87] Karpathiou G., Stefanou D., Froudarakis M.E. (2015). Pleural neoplastic pathology. Respir. Med..

[88] Karpathiou G., Péoc’H M., Sundaralingam A., Rahman N., Froudarakis M.E. (2022). Inflammation of the Pleural Cavity: A Review on Pathogenesis, Diagnosis and Implications in Tumor Pathophysiology. Cancers.

